# Supplementary material for: The Prevalence of Human Plasmodium Species during Peak Transmission Seasons from 2016 to 2021 in the Rural Commune of Ntjiba, Mali
Source: Trop Med Infect Dis. 2023 Sep 7;8(9):438. doi: 10.3390/tropicalmed8090438 (PMC10535850; doi:10.3390/tropicalmed8090438)
Supplement: Supplementary file 1 [file tropicalmed-08-00438-s001.zip › tropicalmed-2536914-supplementary.pdf]

## Supplementary figures

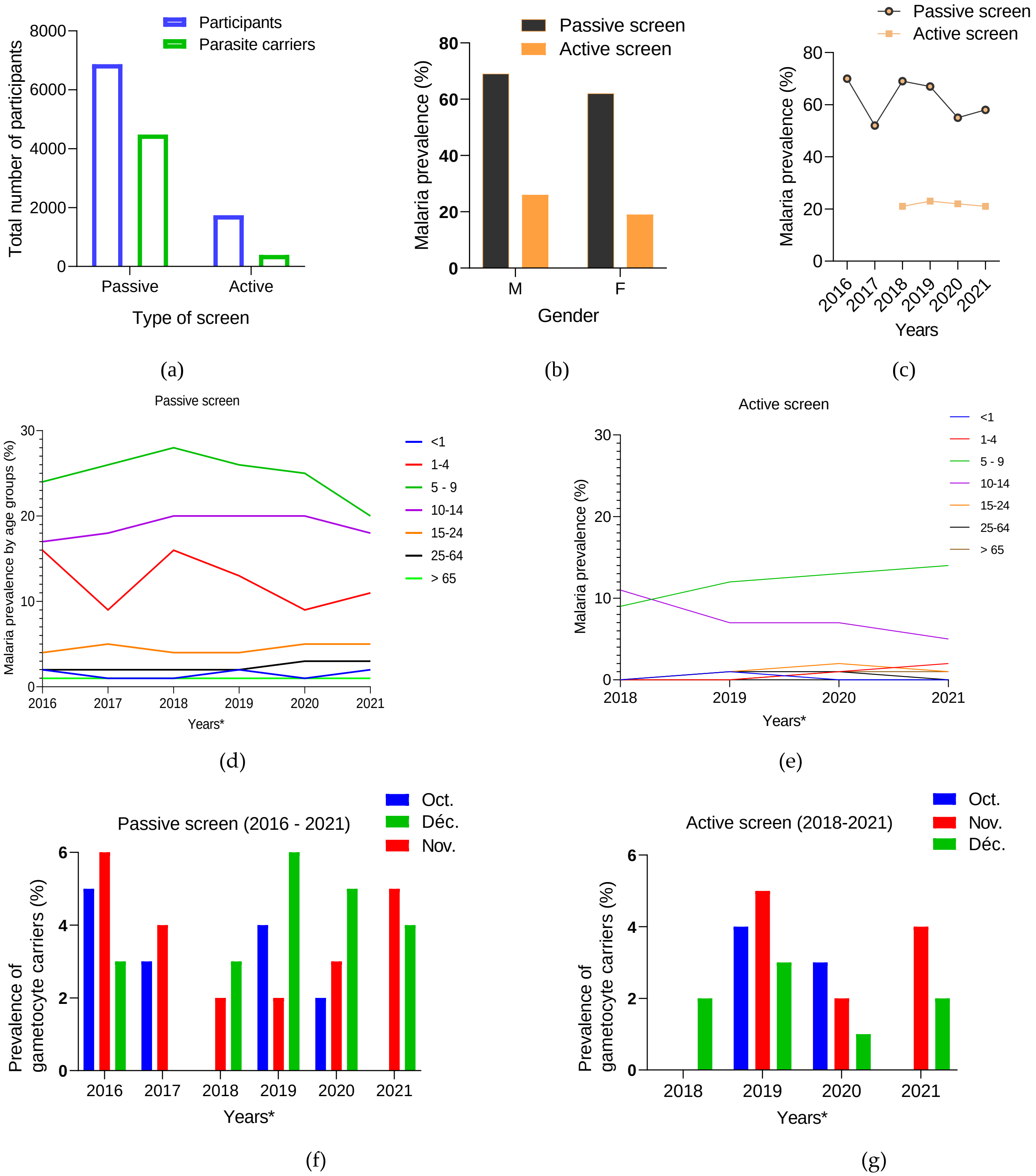


*Indicate that data only cover three months per year (October, November, and December)

**Figure S1.** Distribution of the participants, malaria prevalence per age groups, and by month over the years. In (**a**). we have the total number of participants (blue) and parasite carriers (green) detected in passive and active screen. In (**b**). the prevalence of parasite carriers detected in passive

screen (black) and active screen (orange) by gender of participants. Malaria prevalence by year in symptomatic patients (passive screen) and asymptomatic carriers (active screen) in (**c**). Malaria prevalence distributed by age groups in passive screen in (**d**) and active screen in (**e**). According to the year and the month, we have the prevalence of gametocyte carriers detected in malaria symptomatic patients (passive screen) in (**f**) and asymptomatic carriers (active screen) in (**g**).

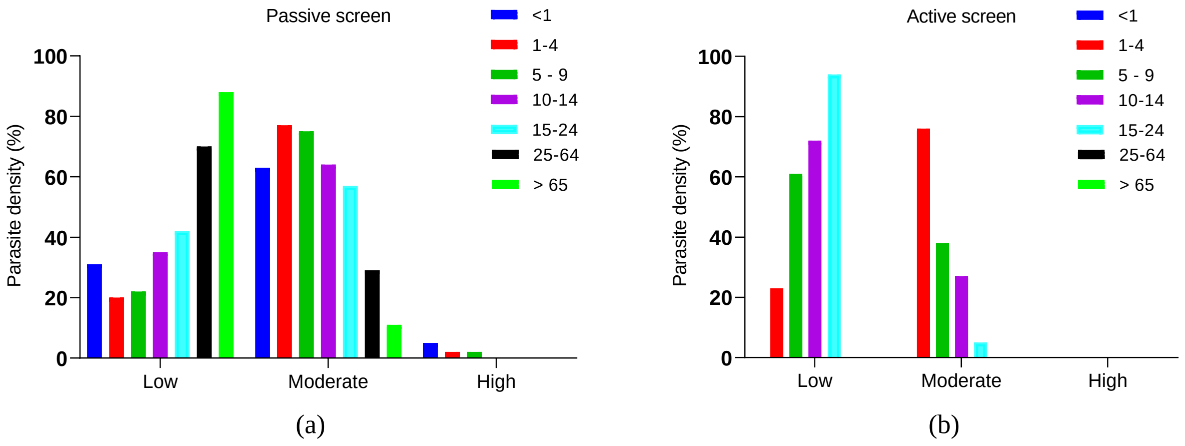


**Figure S2:** The bar chars represent the percentage of parasite densities classified as low, moderate, and high of all *Plasmodium* species detected in symptomatic patients (passive screen, **a**), And the percentage of each parasite densities in asymptomatic parasite carriers (active screen, **b**).
